# Newcastle Disease Virus V Protein Targets Phosphorylated STAT1 to Block IFN-I Signaling

**DOI:** 10.1371/journal.pone.0148560

**Published:** 2016-02-09

**Authors:** Xusheng Qiu, Qiang Fu, Chunchun Meng, Shengqing Yu, Yuan Zhan, Luna Dong, Cuiping Song, Yingjie Sun, Lei Tan, Shunlin Hu, Xiaoquan Wang, Xiaowen Liu, Daxin Peng, Xiufan Liu, Chan Ding

**Affiliations:** 1 Shanghai Veterinary Research Institute, Chinese Academy of Agricultural Sciences, Minhang, Shanghai, China; 2 Key Laboratory of Animal Infectious Diseases, Yangzhou University, Yangzhou, Jiangsu, China; 3 Jiangsu Co-innovation Center for Prevention and Control of Important Animal Infectious Diseases and Zoonoses, Yangzhou, China; University of St Andrews, UNITED KINGDOM

## Abstract

Newcastle disease virus (NDV) V protein is considered as an effector for IFN antagonism, however, the mechanism remains unknown. In this study, the expression of STAT1 and phospho-STAT1 in cells infected with NDV or transfected with V protein-expressing plasmids were analyzed. Our results showed that NDV V protein targets phospho-STAT1 reduction in the cells depends on the stimulation of IFN-α. In addition, a V-deficient genotype VII recombinant NDV strain rZJ1-VS was constructed using reverse genetic technique to confirm the results. The rZJ1-VS lost the ability to reduce phospho-STAT1 and induced higher expression of IFN-responsive genes in infected cells. Furthermore, treatment with an ubiquitin E1 inhibitor PYR-41 demonstrated that phospho-STAT1 reduction was caused by degradation, but not de-phosphorylation. We conclude that NDV V protein targets phospho-STAT1 degradation to block IFN-α signaling, which adds novel knowledge to the strategies used by paramyxoviruses to evade IFN.

## Introduction

Newcastle disease (ND) is one of the most serious and highly contagious diseases of birds and has caused large losses to the poultry industry worldwide every year since 1920s [1]. Its causative agent, Newcastle disease virus (NDV), belongs to the genus *Avulavirus* within the family *Paramyxoviridae* [2]. Newcastle Disease Virus (NDV) includes three types of negative-sense single-stranded, non-segmented genomic RNA at the sizes of 15,186, 15,192 and 15,198 nucleotides (nt) [3–5], and contains six viral genes encoding six structural proteins: nucleocapsid protein (NP), phosphoprotein (P), matrix protein (M), fusion protein (F), haemagglutinin-neuraminidase (HN) and large protein (L) [6].

The P gene of paramyxoviruses encodes three or more viral proteins via RNA editing mechanism [7, 8]; in NDV, the V and W proteins are expressed in addition to P protein expression [9, 10]. In general, there is some probability that one or two non-templated G nucleotides are inserted at position 484 in the ORF of NDV P gene-derived transcripts [10]. The mRNA without frameshift encodes P ORF, and the mRNAs with a +1 or +2 frameshift encode V or W proteins respectively. The three P-gene-encoded proteins shared the same N-terminal region and vary at the C-terminal region [11–14]. The reported proportions of P/V/W protein-encoding mRNAs in NDV-infected cells are 68% for P protein, 29% for V protein and 2% for W protein [15].

Of the three P-gene products, P protein is an essential component of viral RNA-dependent RNA polymerase [6, 16, 17]. The second P-gene-derived product, V protein, is an alpha/beta interferon (IFN-α/β) antagonist, which contributes to viral virulence. Several V-deficient NDV mutants have been recovered using a reverse genetics system for NDV strains Clone-30, Hitchner B1 and Beaudette C to determine the V protein function [15, 18–20]. These V-deficient NDV mutants were more sensitive to the antiviral effects of IFN; resistance was restored when V protein was re-expressed in infected cells. Further studies found that only V protein, but not P and W proteins had IFN-antagonist activity, suggesting that the IFN-inhibitory function of NDV lies in the C-terminal domain (CTD) of the V protein, which promotes degradation of signal transducer and activator of transcription 1 (STAT1) and blocks IFN signaling [19, 21].

STAT proteins are critical mediators of IFN activity. In response to stimulation by IFNs, latent cytoplasmic STAT proteins are phosphorylated on tyrosine by the Janus family of tyrosine kinase (JAK) enzymes. IFNs are commonly classified into two types, IFN-I and IFN-II: the former includes IFN-α/β and the latter includes IFN-γ. Different types of STATs have distinct functions in IFN-I/II signaling. IFN-I is a heterotrimer of phosphorylated STAT-1, STAT-2 and IRF-9; it translocates to the nucleus and binds to cis-acting DNA elements to activate the IFN response. IFN-II is a homodimer of phosphorylated STAT-1 that translocates into the nucleus and binds different cis-acting elements [22, 23].

The detailed mechanism by which V antagonizes the IFN signal has not yet been explained. The above-mentioned theory does not explain the results of Jang et al. [24], who established stable DF-1 cell lines expressing NDV V protein. Stably expressed V protein from either lentogenic or velogenic NDV strains facilitates NDV production, however, the STAT1 protein is still highly expressed and coexists with massive amounts of V proteins in cells. These results suggest that there is a more complicated mechanism in NDV infected cells by which V protein inhibits the IFN signaling. Therefore, the purpose of this study was to investigate the mechanism of NDV antagonizing IFN signaling by measuring the expression of STAT1 and phosphorylated STAT1 (phospho-STAT1) on cells infected with NDV or transfected with plasmids expressing V protein. A reverse-genetics system was used to introduce a stop codon following the editing site of the V gene of the virulent NDV strain ZJ1. Our results demonstrated that NDV V protein targets phospho-STAT1 degradation to block IFN-α signaling.

## Materials and Methods

### Animal ethics

This study was carried out in strict accordance with the recommendations in the Guide for the Care and Use of Laboratory Animals of Shanghai Veterinary Research Institute (SHVRI), the Chinese Academy of Agricultural Sciences (CAAS). BALB/c mice were purchased from Shanghai SIPPR-BK Laboratory Animals Co., Ltd (China). specific-pathogen-free (SPF) chickens were hatched from embryonated SPF chicken eggs (Beijing Merial Vital Laboratory Animal Technology Co., Ltd). The protocols were approved by the Institutional Animal Care and Use Committee (IACUC) of SHVRI, CAAS (Permit Number: shvri-mo-0020 and shvri-po-0028). All surgery was performed under sodium pentobarbital anesthesia, and all efforts were made to minimize suffering.

### Viruses and cells

Lentogenic NDV strain LaSota was obtained from the China Institute of Veterinary Drug Control (Beijing, China). Genotype VII NDV strain ZJ1 was isolated and identified in the Key Laboratory of Animal Infectious Diseases, Yangzhou University [25]. The virulent class I NDV variant 9a5b was obtained from Professors Toshihiro Ito and Koichi Otsuki at Tottori University, Japan [26]. All viruses were grown in 9-day-old SPF embryonated eggs, which were chilled at -80°C for 20 min before the fresh allantoic fluid were taken at fixed time intervals.

Human cell lines A549 and Vero, as well as the chicken fibroblast cell line DF-1 were purchased from ATCC (Manassas, VA, USA). All cells were maintained in Dulbecco’s modified Eagle’s medium (DMEM, GIBCO, Grand Island, NY, USA) supplemented with 10% fetal bovine serum (FBS, GIBCO).

BSR T7/5 cells expressing stably the phage T7 RNA polymerase, was originally established by Buchholz et al. [27], was obtained as a gift from Dr. Zhigao Bu (Harbin Veterinary Institute, Chinese Academy of Agricultural Sciences, China). Cells were maintained in DMEM (GIBCO, Grand Island, NY, USA) supplemented with 10% FBS and 1 mg/mL G418 (Sigma, Saint Louis, MO, USA).

### Construction of V protein-expression plasmids

The P gene open reading frame (ORF) was PCR-amplified from NDV strain LaSota, 9a5b or ZJ1, and inserted into the mammalian expression vector plasmid pCI-neo (Promega, USA) to construct pCI-P/LaSota, pCI-P/9a5b, or pCI-P/ZJ1 [6, 25]. Since P and V mRNAs are almost identical with the only exception of one G insertion at the editing site, the V gene ORFs were established using site-directed mutagenesis to add one nontemplate G residues following the conserved RNA edit motif (5’-AAAAAGGG-3’) in the P gene ORF. PrimeSTARTM HS DNA Polymerase (TakaRa) was used with primer sets mZVF/mZVR, mLVF/mLVR and m9VF/m9VR (Table 1). PCR was performed for 20 cycles of 98°C 10 s, 55°C 15 s and 72°C 7 min. PCR products were purified by PCR purification kits (Axygen) and digested with *Dpn*I (Fermentas) at 1 U/μL at 37°C for 1 h. After inactivation at 80°C for 10 min, 1 μL of digested PCR products was transformed into *E*. *coli* DH5α. All the plasmids were identified by sequencing (Sangon Biotechnology, Shanghai, China), and positive plasmids were designated pCI-V/LaSota, pCI-V/9a5b and pCI-V/ZJ1 respectively.

**Table 1:** Primers used for plasmids construction.

Primers	Primer sequence	Genes
**mZVF**	5' ATC GTC CAA TGC TAA AAA GGG GCC CAT GGT CGG GTT CC 3'	V protein of ZJ1
**mZVR**	5' AAC CCG ACC ATG GGC CCC TTT TTA GCA TTG GAC GAT 3'	
**mLVF**	5' CAA TGC TAA AAA GGG GCC CAT GGT CGA GCC CCC AAG AG 3'	V protein of LaSota
**mLVR**	5' TTG GGG GCT CGA CCA TGG GCC CCT TTT TAG CAT TGG A 3'	
**m9VF**	5' ATC GAA TGC TAA AAA GGG GCC CAC CTC AGA GCC CTC CA 3'	V protein of 9a5b
**m9VR**	5' GAG GGC TCT GAG GTG GGC⋅CCC TTT TTA GCA TTC GAT 3'	
**p9PNF**	5' GGT GAA TTC ATG GCT ACG TTC ACG GAC GCA GAG A 3'	V protein of 9a5b
**p9VCR**	5' CAA TCC GAG TCA ATC TTG CTT ACC CTC TGT G 3'	
**ZJVCDF**	5' A TCA GAA TTC ATG GTC GGG TTC CCA AGA AGG GCA TCA CCA AC 3'	C-terminal domain of ZJ1 V
**pZJVR**	5' CTG ATG A GTC GAC TTA CTT ACC TTC TGT GAT AAT GCC TCC A 3'	
**Fla14sta1F**	5' AGGGATATCGATGTCTCAGTGGTACGAACTTCAGCAGCT 3'	Human STAT1α and its mutant
**Fla14sta1R**	5' TCATGGATCCTACTGTGTTCATCATACTGTCGAATTCTA 3'	

Note: To construct V ORFs, an extra one G was inserted behind the RNA-editing site of pCI-P/ZJ1, pCI-P/LaSota and pCI-P/9a5b using primer sets mZVF/mZVR, mLVF/mLVR and m9VF/m9VR. The added extra G was boxed. To produce anti-V mouse polyclonal serum, the complete V ORF and CTD sequence were PCR-amplified from pCI-V/9a5b and pCI-V/ZJ1 constructed with primer sets p9PNF/ p9VCR and ZJVCDF/ pZJVR. Fla14sta1F/Fla14sta1R were used for PCR amplification of the complete ORFs of *wt* or mutated human STAT1 protein from plasmids stat1 alphap RC/CMV and stat1 alphap Y701F RC/CMV. The restriction endonuclease sites were underlined.

Plasimids stat1 alphap RC/CMV and stat1 alphap Y701F RC/CMV, containing the human STAT1α protein and STAT1α mutant with a Y to F mutation at the 701 aa of phosphorylation site [28] were purchased from Addgene, a nonprofit plasmid repository (http://www.addgene.org/). The complete ORFs of wild-type (*wt*) and mutated human STAT1 protein were PCR-amplified with primers Fla14sta1F and Fla14sta1R (Table 1) and inserted into commercial eukaryotic expression plasmid p3xFLAG-CMV-14 (Siga-Aldrich, USA), and named as pFlag-STAT1 and pFlag-Y701F. The plasmids were verified by sequencing in Sangon Biotechnology (Shanghai, China).

### Preparation of mouse sera against NDV V protein

To produce anti-V mouse polyclonal serum, the complete V ORF and CTD sequence were PCR-amplified from pCI-V/9a5b and pCI-V/ZJ1 constructed with primer sets p9PNF/ p9VCR and ZJVCDF/ pZJVR (Table 1). After double digestion with *EcoR*I and *Xho*I, PCR products were inserted into expression vector pET-28a(+) (Novagen) with an N-terminal His tag in front. Plasmids were transformed into *E*. *coli* DH5α and transformants screened by PCR.

The V protein of 9a5b and the CTD polypeptide from ZJ1 V protein were separately expressed as fusion proteins with 6-histidine tags in *E*. *coli* BL21 as described by the pET system manual. Cultures were grown at 37°C to A_600_ around 0.6 in 2×YT medium (1 L medium containing 16 g tryptone, 10 g yeast extract and 5 g NaCl, pH 7.0 adjusted with NaOH) supplemented with ampicillin. Bacteria were induced by addition of isopropyl-β-d-thiogalactoside (Sigma) at a final concentration of 1 mmol/L. Expression of recombinant proteins was confirmed by Western blot using anti-histidine monoclonal antibody (Mab) (Abcam) and purified by affinity chromatography using a nickel-nitrilotriacetic acid column according to the manufacturer’s instructions (Novagen).

Polyclonal antiserum against the entire V protein or the CTD polypeptide was raised in 6-week-old BALB/c mice. As an antigen, purified recombinant protein was emulsified with an equal volume of Freund’s complete adjuvant (Sigma, USA) and injected subcutaneously into mice followed by three booster shots at two-week intervals. Three days after the last injection, blood was collected, clarified by overnight incubation at 4°C and centrifuged at 4000 rpm for 10 min. Serum was prepared and stored at -20°C. In the course of experiments, mice were monitored twice a day. The food and water were placed in cages for them to take easily. The soft and clean bedding, quiet environment as well as circadian light were provided to reduce animal stress. To minimize animal suffering and distress, all invasive manipulations were carried out under anesthesia by using 1% sodium pentobarbital at a dose of 50 mg/kg. No unexpected death occurred during this study. When the study was completed, mice were euthanized by CO_2_ inhalation for 5 min.

### IFN stimulation tests

To detect STAT1 during infection, A549 and Vero cells were advance-infected with NDVs at a MOI of 3 diluted with DMEM. For infection, cells were cultured in 6-well plates, washed 3 times with PBS, and incubated with NDVs in 600 μL DMEM per well at 37°C for 30 min. Supernatant was discarded and cells were cultured in DMEM containing 2% FBS (Gibco). At indicated time points post infection, cells were stimulated using 500 U/mL human IFN-α or IFN-γ (Sigma) in 1 mL DMEM at 37°C for 15 min. After stimulation, cells were lysed and subjected to Western blot for STAT1 and STAT2. Uninfected cells were stimulated with IFN and tested as negative controls.

To analyze the influence of viral proteins on infection and STAT1 phosphorylation, plasmids encoding P/V proteins or domains were transfected into cells ahead of infection. For transfection, cells cultured in 6-well plates were washed 3 times by PBS and transfected with plasmids or empty plasmid (4 μg/well) using Lipofectamine TM 2000 (Invitrogen, Carlsbad, CA, USA) according to the manufacturer’s protocol. Supernatants were washed out and cells were cultured in DMEM containing 2% FBS (Gibco). At 48 h post transfection, cells were used for infection and IFN-stimulation as above-described.

### Ubiquitination test

To assess whether the ubiquitin-proteasome system (UPS) was involved in degradation of STAT1 during NDV infection, the Ub E1 inhibitor PYR-41 (Roche) was added to media [29]. A WST-1 viability test was performed after 24 h incubation of A549 cells with the inhibitor to determine the concentration of drug to use, according to a standard protocol [30]. After pretreatment with DMEM containing 10% FBS and 10 μg/mL Ub E1 inhibitor at 37°C for 2 h, cells were used for infection, transfection and IFN-stimulation tests as above-described. Ub E1 inhibitor was maintained in media to a final concentration of 10 μg/mL.

### Assay for exogenous STAT1 degradation mediated by NDV

To assess whether NDV target the phosphrylated STAT1 rather than the total STAT1, pFlag-STAT1 and pFlag-Y701F were constructed as described above. The plasmid pFlag-STAT1 encoded a flag-tagged STAT1 protein; while pFlag-Y701F expressed a flag-tagged dominant-negative Stat1α mutant, in which the Tyr-701 was replace by Phe [31]. A549 cells cultured in 6-well plates were transfected with 1 μg pFlag-STAT1 or pFlag-Y701F. At 12 h post-transfection, the cells were subsequently infected with NDVs at a MOI of 3, which was diluted with DMEM, and then cultured in DMEM containing 2% FBS (Gibco) for 24 h. Finally, the cells were lysed and subjected to Western blot for exogenous and total STAT1 with anti-Flag and anti-STAT1 antibodies. Uninfected or untransfected cells were taken as negative controls.

### Western blot

Western blot was performed as described previously [32]. Cells harvested at indicated time points were washed three times with PBS and lysed with RIPA buffer (50 mM Tris-HCl pH 7.6, 150 mM NaCl, 1% v/v NP-40, 1% w/v sodium deoxycholate, 0.1% w/v sodium dodecyl sulfate, 1 mM phenylmethane sulphonylfluoride, 1 mM Na3VO4, 1 mM NaF, 0.15 μM aprotinin, 1 μM leupeptin, and 1 μM pepstatin). Equal amounts of cell extracts were separated by SDS-PAGE on a 7.5% gel and transferred to nitrocellulose membranes. Total STAT1 and phosphorylated STAT1 was detected using anti-STAT1 antibody (ab31369) and anti-phospho-STAT1 antibody (ab30645) purchased from Abcam (MA, USA). Mouse monoclonal anti-β-actin antibody (A1978) was from Sigma-Aldrich. Protein-antibody interactions were detected using goat anti-mouse IgG conjugate (Sigma).

### Immunofluorescence

A549 and Vero cells were seeded on glass coverlips and then transfected with V protein expression plasmids or transfected with NDV as described above. At 48 h post-transfection or 12 h post-infection, they were fixed and permeablized in ice-old methanol. For analysis of the phosphorylated STAT1, a half of coverlips were treated with 500 U/mL human IFN-α for 15 min in serum-free DMEM before fixation. Fixed cells were probed for P/V proteins with a mixture of anti-serum Pab-V1 and Pab-V2; while the presence of STAT1 and phosphorylation were determined by anti-STAT1 antibody (ab31369) and anti-phospho-STAT1 antibody (ab30645). Cellular nuclei were stained with 4,6-diamidino-2-phenylindole (DAPI). After incubation with secondary antibodies, the cells were observed under fluorescence microscopy.

### Recovery of recombinant virus

Construction of plasmid pNDV/ZJ1 containing the full-length cDNA of the class I genotype VIId strain ZJ1 was described previously [25, 33]. The entire genome of ZJ1 flanked by the T7 promoter sequence and the hepatitis delta virus ribozyme sequence was inserted into the vector pTVT7R(0.0). After transcription with T7 polymerase in BSR T7/5 cells, genomic RNA is an exact copy of the *wt* NDV genome [33, 34].

To construct a V-deficient NDV genome, the C-terminal region of P gene was PCR-amplified with primers PVStop (5’-*GGG CCC*TAG GTC GGG TTC CCA AGA AG-3’) and PZJPAR (5’-*GGG CCC* CCG AGG GAT TCC GTG T-3’) from plasmid pNDV/ZJ1 to introduce an AT to TA mutation. Mutated PCR product was digested with *Apa*I and ligated into plasmid pTX37, constructed from *Bgl*II-digested pNDV/ZJ1. The resulting plasmid was double-digested with *Xba*I and *BstZ*17I and ligated into pNDV/ZJ1. The recombinant plasmid carrying V-deficient genome of ZJ1 was designated pNDV/ZJ1-VS.

Recovery of infectious recombinant virus from pNDV/ZJ1-VS followed procedures described previously [25, 33]. Plasmids pNDV/ZJ1-VS and mixture of three expression plasmids encoding NP, P, and L proteins of ZJ1 were transfected into BSR T7/5 cells. After inoculation of 10-day-old embryonated SPF chicken eggs with transfected culture supernatant, the infectious recombinant NDV rZJ1-VS strain was harvested from fresh allantoic fluid. HA tests were performed in 96-well microtiter plates to determine the HA titer of allantoic fluids containing recombinant virus.

Genomic RNA of rZJ1-VS was extracted from fresh allantoic fluid using TRIzol reagent (Invitrogen) and cDNA was reverse transcribed with 6-nt random primer. With primers covering the entire viral genome, recombinant viruses were PCR amplified from cDNA as described [25, 33]. Aliquots of PCR products were analyzed on 1% agarose gels. PCR products were purified using PCR purification kits (Axygen, USA) and sequenced by Sangon Biotechnology (Shanghai, China).

### Characterization of recovered rZJ1-VS strain

MDT, ICPI, and IVPI of recovered rZJ1-VS virus and its *wt* virus ZJ1 were measured according to standard procedures [35, 36]. Growth characteristics and CPE of recombinant viruses were compared with *wt* viruses on DF1 and Vero cells. Confluent monolayer cells were infected at MOI 0.01 and incubated at 37°C in DMEM containing 2% FBS. CPE caused by NDV was observed at 12 h intervals. Supernatants were harvested and tittered by plaque assays on DF1 cells. Assays were repeated three times and results were analyzed and displayed in growth curves.

### Enzyme-linked immunosorbent assay (ELISA) for IFN-β

A549 cells seeded into 6-well plates were infected with ZJ1-VS or *wt* ZJ1 virus. The cells without virus infection were used as controls. The culture supernatant (1.5 mL) were harvested at 2, 4, 6, 12, 24 h post-infection and assayed for IFN-β using VeriKine Human IFN Beta ELISA kit (Pestka Biomedical Laboratories) following the manufacturer’s protocol. The expression levels of IFN-β protein (pg/mL) stimulated by NDV were calculated based on a standard curve generated at the time of the assay.

### Real-time quantitative polymerase chain reaction (PCR) assay

Real-time qRT-PCR was performed to measure the mRNA levels of IFN downstream antiviral genes, including IFIT1, OAS1, ISG15, Mx1, and IFN-β genes. Human actin gene was used as the endogenous control. First of all, a monolayer of A549 cells was washed with PBS three times and infected with NDV ZJ1 or rZJ1-VS at a MOI of 3. At 6 h post-infection (hpi), cells were washed with PBS and harvested in 1 mL TRIZol reagent (Invitrogen, Carlsbad, CA, USA). Alternatively, A549 cells in 6-well plates were transfected with 3 μg pCI-V or pCI-neo for each well as above-described. At 4 h and 8 h post-transfection, those cells were harvested in 1 mL TRIZol reagent (Invitrogen, Carlsbad, CA, USA) following by the treatment with 500 U/mL IFN-α for 30 min.

The total RNA was extracted as described above, and then transcribed into cDNA by M-MLV reverse transcriptase (Promega, Madison, WI, USA) with the 6-nt random primer. Real-time qRT-PCR was performed using SYBR Premix Ex Taq reagents (Takara, Dalian, China) in the the Mastercycler ep realplex4 apparatus (Eppendorf, Germany). A final volume of 20 μL of PCR reaction system was used, including 1 μL cDNA, 10 μL of enzyme mixture from the kit, and 10 pmol of primer pairs (Table 2) as previous reported [37, 38]. The PCR cycles are as follows: 95°C for 20 s, followed by 40 cycles of 95°C for 10 s, 60°C for 15 s and 72°C for 20 s. At the end of the reaction, melting temperature of the final double-strand DNA product was determined by intercalated SYBR Green. PCR efficiencies were above 1.85 and amplifications generated single expected amplicons with single, sharp fusion curves. All experiments were carried out in triplicate. The changes of mRNAs were presented as fold expression and calculated using the comparative C_T_ (^ΔΔ^C_T_) method as described [39].

**Table 2:** Real-time PCR primers used to measure the mRNA levels of IFN downstream antiviral genes.

Primers	Primer sequence	Genes
hIFIT1L	5' GCC ATT TTC TTT GCT TCC CCT 3'	IFIT1
hIFIT1R	5' TGC CCT TTT GTA GCC TCC TTG 3'	
hOAS1L	5' CAT CCG CCT AGT CAA GCA CTG 3'	OAS1
hOAS1R	5' CCA CCA CCC AAG TTT CCT GTA G 3'	
hISG15L	5' CTT TGC CAG TAC AGG AGC TT 3'	ISG15
hISG15R	5' GCC CTT GTT ATT CCT CAC CA 3'	
hMX1L	5' AAT CAG CCT GCT GAC ATT GG 3'	MX1
hMX1R	5' GTG ATG AGC TCG CTG GTA AG 3'	
hIFNbF	5' TTG TGC TTC TCC ACT ACA GC 3'	IFN-β
hIFNbR	5' CTG TAA GTC TGT TAA TGA AG 3'	
mßactinrtL	5' GAT CTG GCA CCA CAC CTT CT 3'	ß-actin
mßactinrtR	5' GGG GTG TTG AAG GTC TCA AA 3'	

### PCR arrays of IFN-associated factors in DF1 cells

Human Type I Interferon Response RT² Profiler PCR Arrays (Qiagen, PAHS-016Z) containing genes involved in IFN-α/β immune responses were used to determine differences in IFN-associated gene expression between rZJ1-VS and *wt* ZJ1-infected cells. Confluent monolayer A549 cells in 3.5 cm diameter culture dishes were infected with rZJ1-VS and ZJ1 at MOI 3 and incubated at 37°C in DMEM containing 2% FBS. At 8 hpi, cells were lysed in culture dishes with 1 mL TRIzol reagent (Invitrogen, USA). Viral RNA was extracted and cleaned with RNeasy MinElute Cleanup Kits (Qiagen, USA) following the manufacturer’s instructions. Purified RNA was sent to the KANGCHEN Biotechnology Company (Shanghai, China) for PCR assays. Raw Ct values were analyzed using RT^2^ Profiler PCR Array Data Analysis. (http://pcrdataanalysis.sabiosciences.com/pcr/arrayanalysis.php?target=upload).

## Results

### NDV V protein did not function on STAT1 degradation efficiently

**Fig 1:**
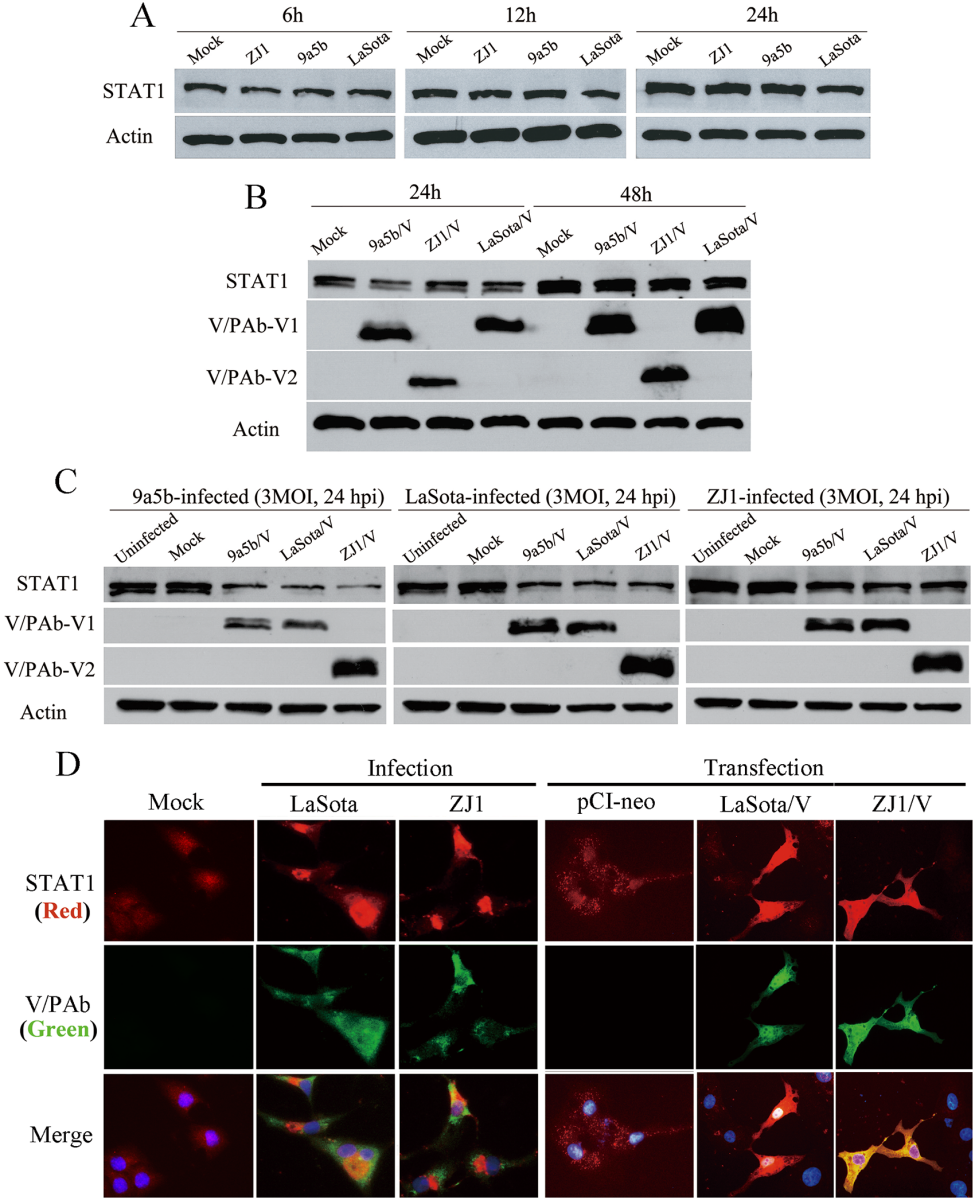
STAT1 expression in NDV infected or V-expressing plasmids transfected cells. (A) No STAT1 reduction was observed in Vero cells infected with NDV ZJ1, 9a5b or LaSota at MOI 3 at 6, 12 or 24 hpi. (B) Over-expression of ZJ1, 9a5b and LaSota V protein did not effect on STAT1 expression in Vero cells transfected with V-expressing plasmids. (C) STAT1 was reduced at 48 h post-transfection in NDV-infected Vero cells (MOI = 3) transfected in advance with V-expressing plasmids. (D) STAT1 expression in A549 cells transfected with V expressing plasmids or infected with ZJ1 or LaSota was detected at 48 h post-transfection or 12 h post-infection by indirect fluorescence assay. These infected A549 cells were fixed and detected for P/V/W proteins with a mixture of anti-serum Pab-V1 and Pab-V2; while the presence of STAT1 was determined by anti-STAT1 antibody (ab31369). Cellular nuclei were stained with 4,6-diamidino-2-phenylindole (DAPI).

NDV V protein is reported to target STAT1 degradation to block IFN signaling [15, 18–21]. In our study, the Vero cells and A549 cells were infected with NDV at a multiplicity of infection (MOI) of 3, and the STAT1 expression in infected cells was detected by Western blot at different time points post infection (p.i.). Compared to mock-infected cells, no obvious reduction in STAT1 expression was observed in NDV infected Vero cells (Fig 1A). Western blots showed similar levels of STAT1 expression in cells infected with LaSota (genomic RNA of 15,186 nt), ZJ1 (15,192 nt) or 9a5b (15,198 nt) viruses at all the time points detected.

To investigate whether over-expression of NDV V protein had an effect on STAT1 degradation, a series of plasmids were used [25, 40, 41]. The P gene ORF from LaSota, 9a5b and ZJ1 viruses was polymerase chain reaction (PCR)-amplified and ligated into the mammalian expression vector plasmid pCI-neo (Promega, USA). Positive plasmids confirmed by sequencing were designated pCI-V/LaSota, pCI-V/9a5b and pCI-V/ZJ1.

A549 cells were transfected with V-expressing plasmids and harvested for Western blot detection at 24 and 48 hpi. Anti-serum Pab-V1 recognized the V protein of LaSota and 9a5b but did not react with ZJ1 V protein. Pab-V2 antiserum reacted only with ZJ1 V protein. Over-expression of V protein from virulent or nonvirulent NDVs did not lead to marked reduction of STAT1 proteins compared to mock cells (Fig 1B). Only when cells were subsequently infected with LaSota at a MOI of 3 at 48 hpi, the STAT1 proteins were partially degraded (Fig 1C). Similar patterns were observed upon infection with the viruses ZJ1 and 9a5b. Furthermore, the STAT1 levels in NDV-infected A549 cells were analyzed by indirect immunofluorescence assay (IFA) (Fig 1D).

STAT1 was not obviously degraded in NDV-infected A549 cells; however, it seems that STAT1 proteins were sequestrated in the cytoplasm in the course of infection. The significance of co-existence of NDV V protein and STAT1 are not clear yet.

### NDV V protein targets phospho-STAT1 degradation in NDV infected cells

NDV infection or transfection with V-expressing plasmids alone did not strongly affect the level of STAT1, so we analyzed whether STAT1 phosphorylation patterns were altered in NDV-infected cells. Phosphorylation of STAT1 and STAT2 by the JAK family is a prerequisite for activation and expression of IFN-induced genes. We assessed STAT1 phosphorylation patterns in IFN-stimulated A549 cells at 5-min intervals. The results showed that stimulation of 500 U/mL IFN increased phospho-STAT1 in cells (see S1 Fig). However the effect was not long-lasting. At 60 min post stimulation, phospho-STAT1 was undetectable, which might be de-phosphorylated by cells [42, 43]. For this reason, following test was designed to analyze the actual relationship between phospho-STAT1 and NDV infection. Cells infected with NDVs were further treated with subsequently stimulation with 500 U/mL IFN-α for 15 min prior to each time point for harvest.

**Fig 2:**
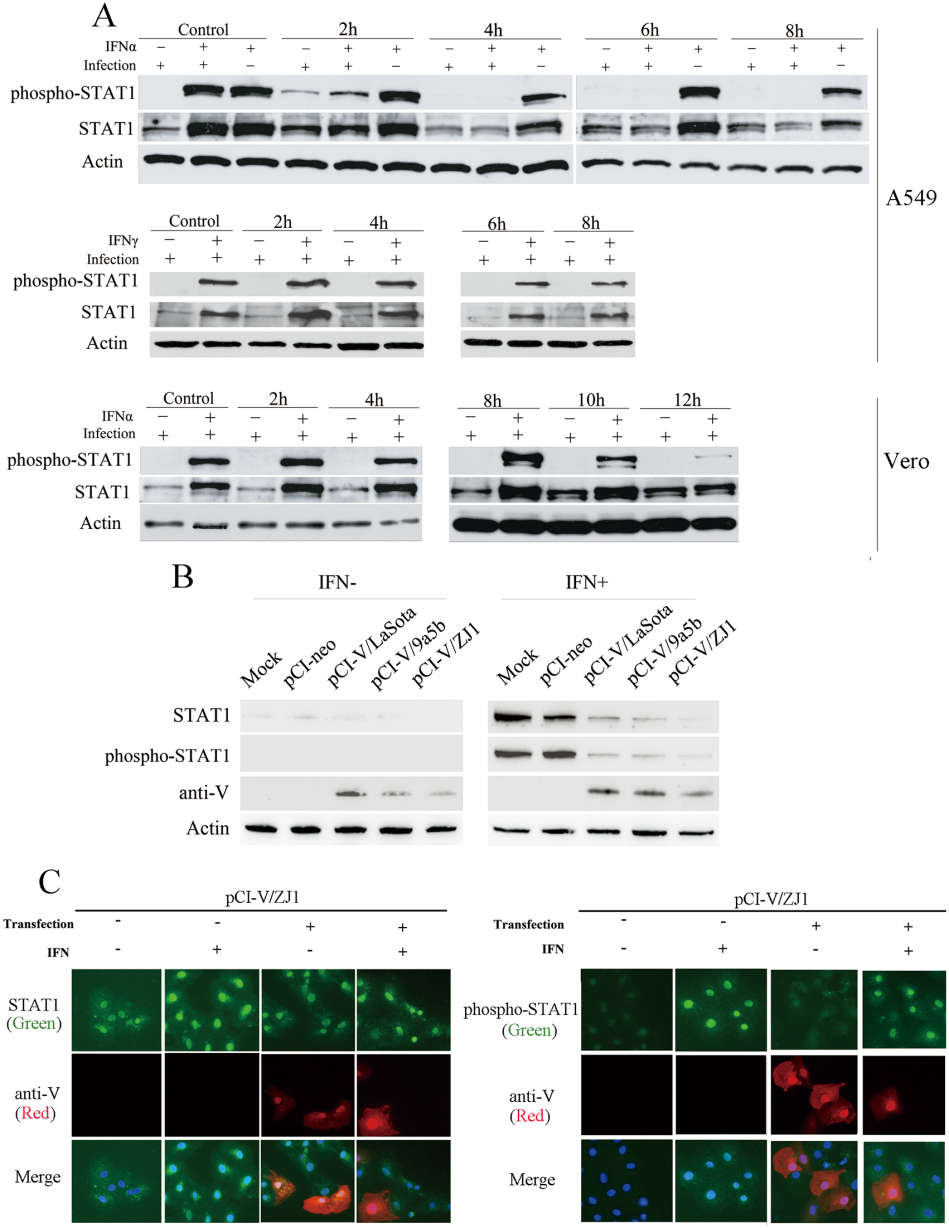
Newcastle disease virus infection impaired IFN-α-induced STAT1 phosphorylation. (A) IFN-α-induced phosphorylated STAT1 degradation in NDV-infected A549 and Vero cells. A549 or Vero cells were infected with NDV strains ZJ1 at MOI 3. At indicated time points post infection, A549 and Vero cells were stimulated with 500 U/ml human IFN-α or IFN-γ in 1 ml DMEM at 37°C for 15 min. Uninfected cells were stimulated with IFN as negative controls (IFNα+/infection-). IFN-α-induced phospho-STAT1 was observed (IFNα+/infection+) for reduction in total STAT1 proteins. IFN-γ-induced phospho-STAT1 proteins were not reduced. (B) Phospho-STAT1 in A549 cells transfected with V-expressing plasmids after stimulation with IFN-α. (C) Expression level of STAT1 and phospho-STAT1 decreased in V-expressing Vero cells after IFN-α stimulation. Vero cells were mock transfected with pCI-neo plasmids. Cells on glass coverlips were transfected with pCI-V/ZJ1 plasmids. At 48 h post-transfection, cells were treated with IFN-α for 15 min prior to fixation as in “Material and methods”. V protein was detected by a mixture of anti-serum Pab-V1 and Pab-V2; STAT1 and phosphorylation were determined by anti-STAT1 antibody (ab31369) and anti-phospho-STAT1 antibody (ab30645). Cellular nuclei were stained with DAPI.

IFN-α-induced phospho-STAT1 expression were largely reduced in NDV-infected A549 cells (designated IFNα+/infection+) at 2 hpi and were undetectable after 4 hpi (Fig 2A); while the STAT1 in uninfected cells was phosphorylated (designated IFNα+/infection-) after stimulation with IFN-α. The level of total STAT1 gradually reduced with the decrease of phospho-STAT1. Indirect effects on phospho-STAT1 reduction caused by virus absorption and entry could be ruled out, as STAT1 expression and phosphorylation in cells examined immediately after incubation with viruses (Mock) were not different from IFN-treated uninfected cells.

We obtained similar results in Vero cells with phospho-STAT1 starting to reduce at 10 hpi. The control of mock-stimulated A549 and Vero cells were treated with phosphate buffer saline (PBS) infected with same virus (designated IFNα-/infection+). Phospho-STAT1 proteins were undetectable after infection, except at 2 hpi in A549 cells. The level of total STAT1 was not noticeably changed. This experiment was repeated with IFN-γ instead of IFN-α. After stimulation, induction of STAT1 was not affected by NDV infection, ruling out the possibility that STAT1 reduction was caused by cellular activities due to ongoing viral replication (Fig 2A).

We showed that NDV infection led to the reduction of phospho-STAT1 in infected cells. To determine the function of V protein in phospho-STAT1 reduction, eukaryotic plasmids expressing V from ZJ1, LaSota and 9a5b viruses were constructed and transfected into A549 cells. At 48 h post transfection, STAT1 was highly stimulated in control cells transfected with pCI-neo after stimulation with IFN-α. By contrast, the STAT1 expression level obviously decreased in cells expressing V proteins from ZJ1, LaSota and 9a5b (Fig 2B). Similar results were observed by IFA, the reduction of STAT1 in V-expressing cells was merely detected after stimulation of IFN-α (Fig 2C). These results suggested that IFN-α-induced phosphorylation precedes STAT1 reduction. The decrease of phospho-STAT1 led to the reduction of STAT1 expression.

**Fig 3:**
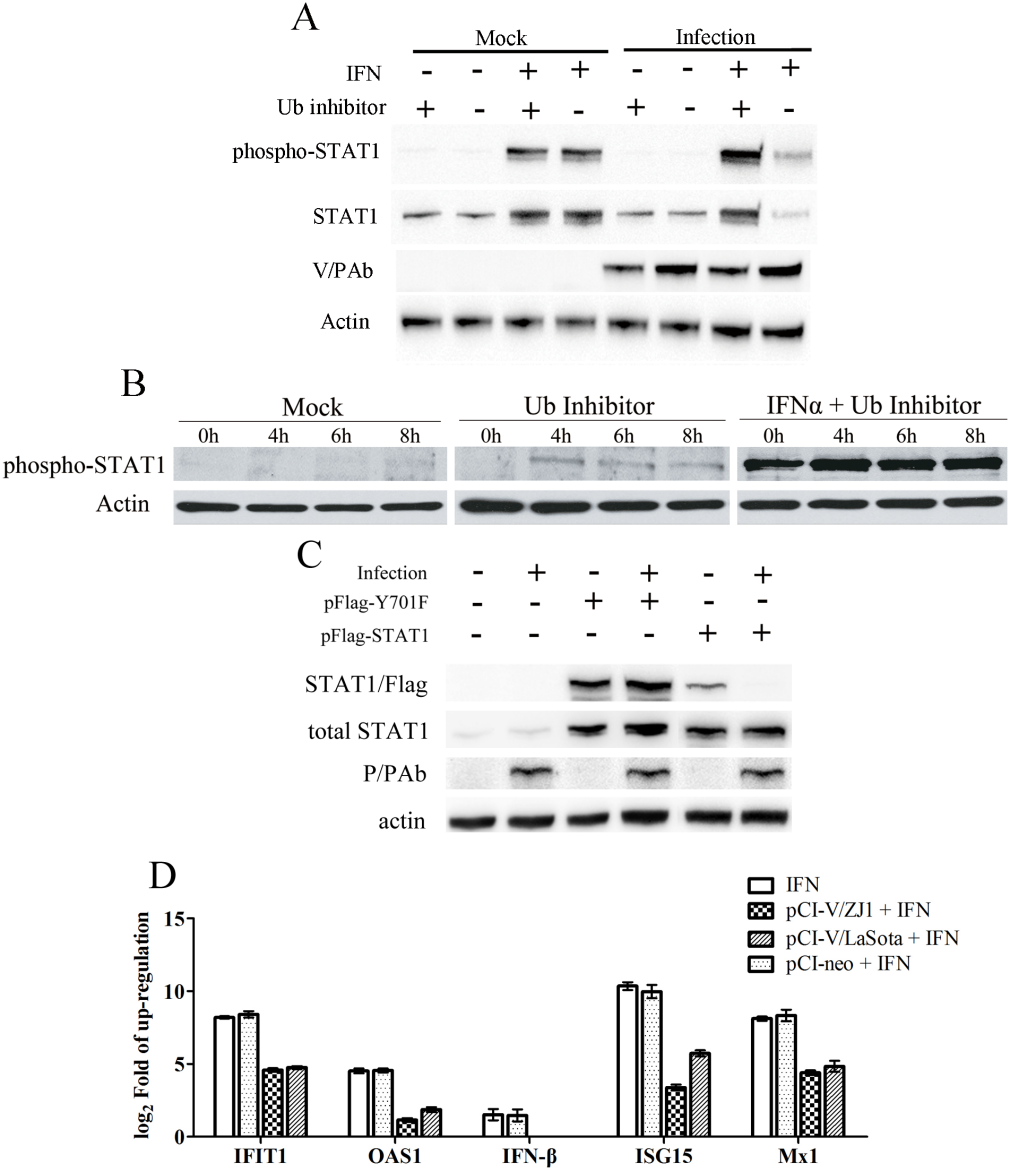
The reduction of total and phosphorylated STAT1 in NDV-infected cells was inhibited after treatment with Ub E1 inhibitor PYR-41 at different time points. (A) STAT1 and phospho-STAT1 expression levels in PYR-41 treated Vero cells at 6 hpi. (B) Phospho-STAT1 levels in PYR-41 treated Vero cells at 4, 6, 8 hpi. (C) Exogenous mutant STAT1 lacking 701aa phosphorylation site were not degraded in the course of NDV infection. One microgram pFlag-STAT1 or pFlag-Y701F was transfected into A549 cells cultured in 6-well plates. At 12 h post transfection, the cells were subsequently infected with NDVs at a MOI of 3. The cells were harvested at 24 hpi. (D) The expression levels of IFN-responsive genes in V-expressing A549 cells after stimulation of IFN-α. A549 cells in 6-well plates were transfected with 3 μg pCI-V or pCI-neo for each well as above-described. At 4 h and 8 h post-transfection, the cells were harvested following the treatment with 500 U/ml IFN-α for 30 min.

To determine whether the reduction of phospho-STAT1 was caused by degradation or dephosphorylation, we analyzed phospho-STAT1 in cells treated with an ubiquitin E1 inhibitor PYR-41 (Roche). PYR-41 acts on the ubiquitin-activating enzyme E1 to block protein degradation mediated by the ubiquitin-proteasome system [29]. When the ubiquitin enzyme was inhibited, phospho-STAT1 was not affected by NDV infection (Fig 3A and 3B), suggesting that reduction of phospho-STAT1 was mediated by degradation rather than de-phosphorylation. Furthermore, plasmids expressing STAT1 protein or mutated STAT1 lacking Y701 phosphorylation site were constructed so as to determine whether NDV target phosphorylated STAT1. After co-infection with NDV, the exogenous STAT1 expressed by plasmids were obviously degraded by NDV. In comparison, the expression level of dominant-negative STAT1 Y701 was not affected (Fig 3C), suggesting the phosphorylated STAT1 was the target for NDV. The expression levels of IFN-β, OAS1, IFIT1, ISG15 and Mx1 in cells expressing V proteins were assayed so as to determine whether the degradation of pSTAT1 mediated by V protein blocks IFN signaling. As shown in Fig 3D, these IFN-responsive genes were obviously up-regulated after stimulated by IFN-α in mock cells and those cells transfected pCI-neo vector. By contrast, IFN signaling were suppressed in cells expressing V protein from LaSota or ZJ1 as the levels of IFN-β, OAS1, IFIT1, ISG15 and Mx1 were obviously lower than that of IFN-stimulated mock cells.

### Rescue of recombinant NDV with absence of V protein C-terminal domain

**Fig 4:**
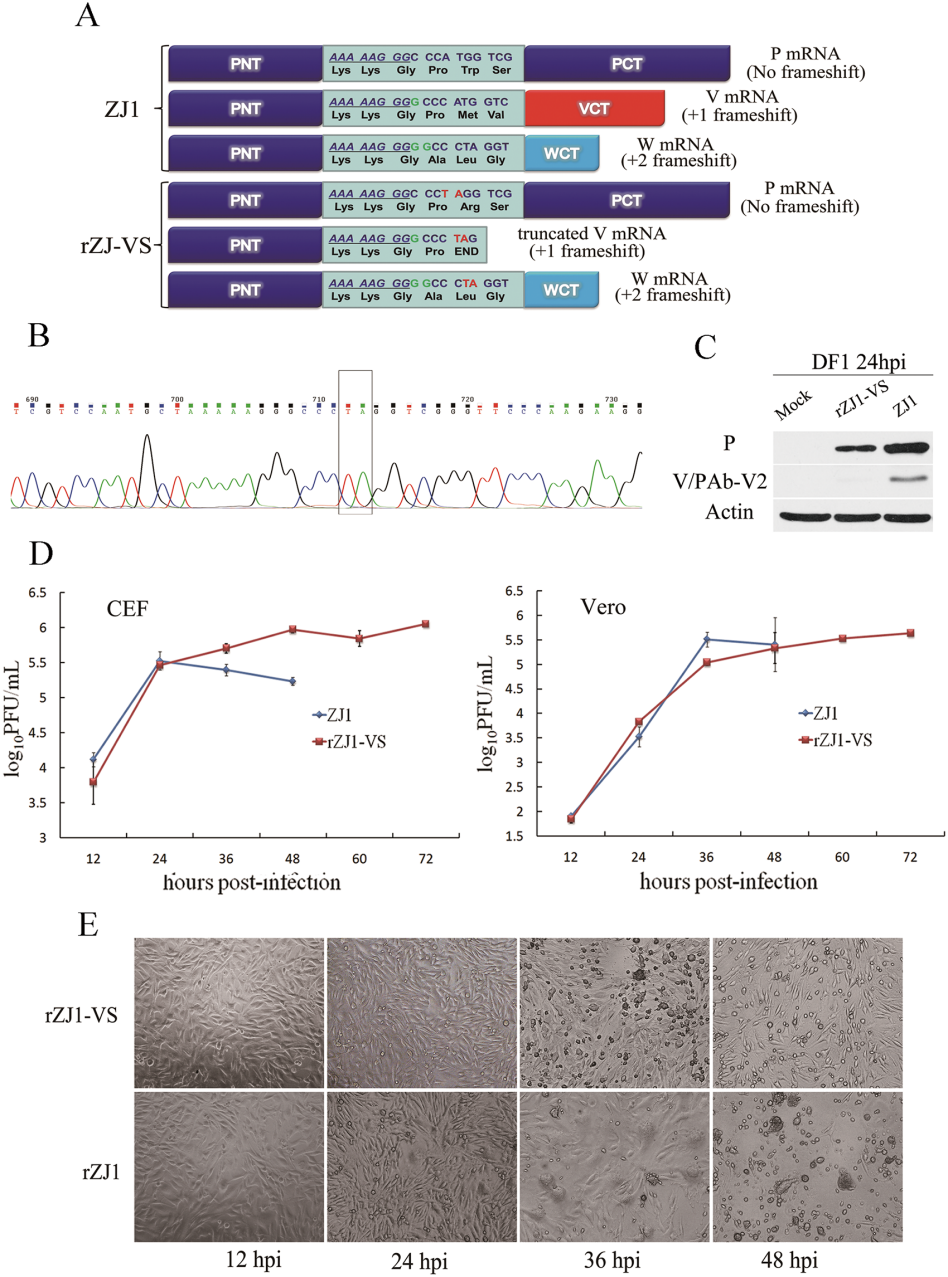
Recovery of V-deficient recombinant NDV from an infectious clone. (A) Schematic representation of a V-deficient gene mutation in a full-length cDNA clone. To selectively block expression of V, modification was performed after the RNA-editing site of the full-length cDNA clone pTVT-ZJ1 to introduce a stop codon in the ORF with +1 frameshift. (B) Mutation in the genome of recovered rZJ1-VS was confirmed by sequencing. Total viral RNA was extracted and RT-PCR-amplified for sequencing. The genome of rZJ1-VS was identical to the *wt* ZJ1 except for AT for TA after the RNA editing-site. (C) Expression of V protein was examined in DF-1 cells infected with rZJ1-VS or ZJ1 at MOI 3. The V protein was undetectable in the rZJ1-VS infected cells; by contrast, ZJ1 expressed V protein in the infected cells at the same point. (D) Growth curves of rZJ1-VS and the *wt* ZJ1 in DF1 and Vero cells. Replication of rZJ1-VS was compared to ZJ1 in early infection. (E) Cytopathic effect (CPE) caused by rZJ1-VS or ZJ1 in DF1 cells infected with rZJ1-VS or ZJ1 at MOI 0.01.

To selectively block V protein expression, modifications were made after the RNA-editing site of the full-length cDNA clone pTVT-ZJ1 by introducing a stop codon in the ORF with +1 frameshift (Fig 4A). The verified V-deficient recombinant full-length cDNA clone plasmid was named pZJ1-VS. Following previous procedures [25], the full-length cDNA clone mutant pZJ1-VS was co-transfected into BSR T7 cells with helper plasmids pCI-NP, pCI-P and pCI-L in the proportion of 1:1:0.5:0.5. At 72 h post transfection, cells were lysed and injected into the allantoic cavity of 10-day-old SPF chicken embryonated eggs, which were dead at 60–96 h post inoculation. The haemagglutination (HA) titers of allantoic fluid were 10^4^–10^5^. After passage in embryonated SPF chicken eggs for two generations, HA titers were 10^7^. Only the third generation of recombinant virus rZJ1-VS was used in following studies.

To confirm the genome mutation of recovered rZJ1-VS, total viral RNA was extracted and analyzed by RT-PCR using primer pairs covering entire viral genome. PCR products were sequenced and compiled into a full-length cDNA of rZJ1-VS. All of three generations of rZJ1-VS contained a genomic RNA of 15,192 nt, which were identical to the *wt* ZJ1 except for the AT for TA mutation in the P gene (Fig 4B).

V protein expression was examined in DF1 cells infected with rZJ1-VS or ZJ1 at a MOI of 3. At 24 hpi, cell lysate was tested for V protein by Western blot with antiserum Pab-V2. V protein was undetectable in rZJ1-VS infected cells; in contrast, ZJ1 expressed V protein in infected cells at the same point, suggesting a V-deficient recombinant NDV was recovered (Fig 4C).

### Biological characteristics of recovered rZJ1-VS

A recombinant NDV mutant expressing deficient V protein was generated from a full-length cDNA clone of genotype VIId NDV. Growth characteristics of rZJ1-VS and *wt* ZJ1 were examined in 10-day-old embryonated SPF chicken eggs. Eggs were inoculated by the allantoic route with 0.2 ml rZJ1-VS or ZJ1 at 10^−5^, 10^−6^ or 10^−7^. The death time of eggs and the HA titer of allantoic fluid were examined. Both viruses killed eggs at 36–60 hpi and accumulated in the allantoic fluid up to an HA titer of 10^7-8^.

The deficiency in the CTD domain of V protein retarded the growth of rZJ1-VS in infected cells. DF-1 cells were infected with rZJ1-VS or ZJ1 at a MOI of 0.01. At 24 hpi, the *wt* virus caused cytopathic effect (CPE) observed as decreased cell diopter, rounding reduced cells, modified particles and formation of multinuclear large cells due to cell fusion. At 36 hpi, large pieces of cells and multinuclear large cells were shedding off or lysed. The monolayer was damaged at 48 hpi with ZJ1 (Table 2 and Fig 4D). Thus, typical CPE was caused by virulent NDV. In contrast, rZJ1-VS caused mild CPE. In late infection, growth of rZJ1-VS was retarded compared to the *wt* viruses and no multinuclear large cells were observed (Fig 4D). CPE was first observed at 36 hpi and monolayer was damaged at 84 hpi, 36 h later than the results with ZJ1. CPE was observed as cell shrinkage, cell size reduction, rounding and shedding off (Fig 4E). No fused cell was observed. Similar results were obtained with Vero cells (Fig 4E). More cells survived post infection of rZJ1-VS at all the time points investigated, with slightly higher viral titers in the supernatant in late infection and prolonged shedding time (Table 3). Retardation of rZJ1-VS in cells did not affect viral pathogenicity. It showed that the recombinant virus, rZJ1-VS was virulent, with a mean death time (MDT) of 45.6, an intracerebral pathogenicity index (ICPI) of 1.74 and an intravenous pathogenicity index (IVPI) of 2.47 (Table 4).

**Table 3:** Cytopathic effects and virus titers of NDV-infected cells.

Cells	Virus	Time of Cytopathic Effect Observed (h)	Time of Monolayer totally Damaged (h)	Highest Titer in Supernatant (log_10_PFU/ml)
**CEF**	rZJ1	24	48	6.51
**CEF**	rZJ-VS	24	84	6.99
**Vero**	rZJ1	24	60	6.58
**Vero**	rZJ-VS	24	96	6.63

**Table 4:** Determination of virus virulence.

Viruses	MDT^a^	ICPI^b^	IVPI^c^
**ZJ1**	48.0	1.89	2.54
**rZJ-VS**	45.6	1.74	2.47

^a^ mean death time

^b^ intracerebral pathogenicity index of 1-day-old chickens

^c^ intravenous pathogenicity index of 6-week-old chickens

### V-deficient rNDV did not lead to degradation of phospho-STAT1

V protein appeared to be important in the degradation of phosphorylated STAT1. To verify this result, a recombinant NDV expressing a truncated V protein lacking the CTD region was generated. The CTD of V protein from NDV contains a cysteine rich domain that is commonly considered to be critical for STAT1 degradation.

**Fig 5:**
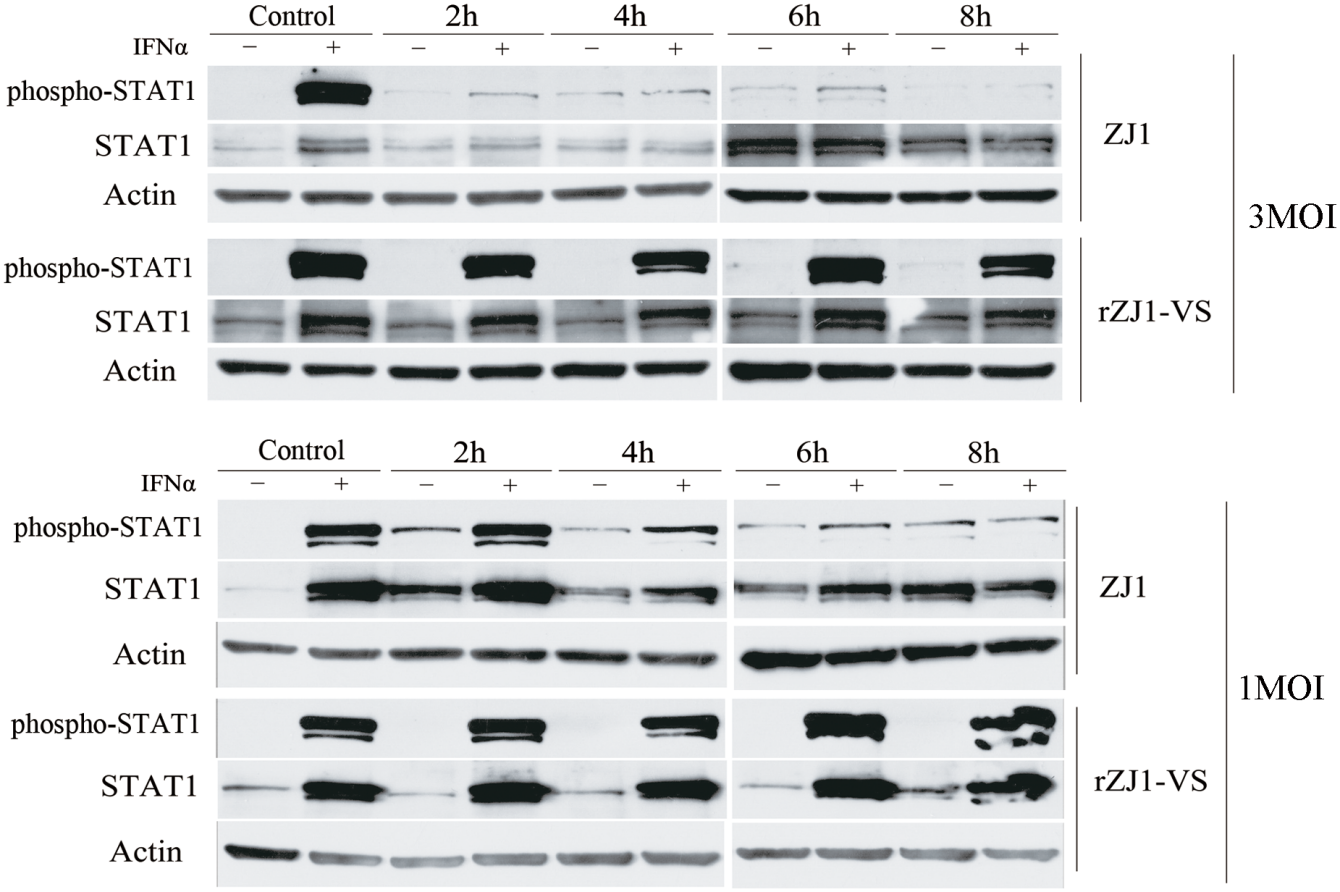
Infection with rZJ1-VS did not result in degradation of phospho-STAT1. A549 cells were infected with rZJ1-VS or *wt* ZJ1 at a MOI of 1 or 3. At indicated time points, cells were stimulated with 500 U/mL IFN-α for 15 min and harvested for Western blot. Phospho-STAT1 and STAT1 was observed in A549 cells infected with ZJ1 or rZJ1-VS, which expressed incomplete V protein.

To determine whether V protein degraded phospho-STAT1, A549 cells were infected with rZJ1-VS or ZJ1 at a MOI of 1 and 3. Cells infected with NDVs were harvested at indicated time points after stimulation with 500 U/ml IFN for 15 min, so STAT1 was phosphorylated as shown in mock-infected cells (Fig 2). Infection by ZJ1 of A549 cells led to obvious reduction of phospho-STAT1 with subsequent reduction of total STAT1 protein (Fig 5). In contrast, no reduction of phospho-STAT1 or STAT1 was observed in cells infected with rZJ1-VS, which expressed a deficient V protein. This result suggested that V protein was critical for phospho-STAT1 degradation.

### IFN-responsive genes in rZJ1-VS infected cells

**Fig 6:**
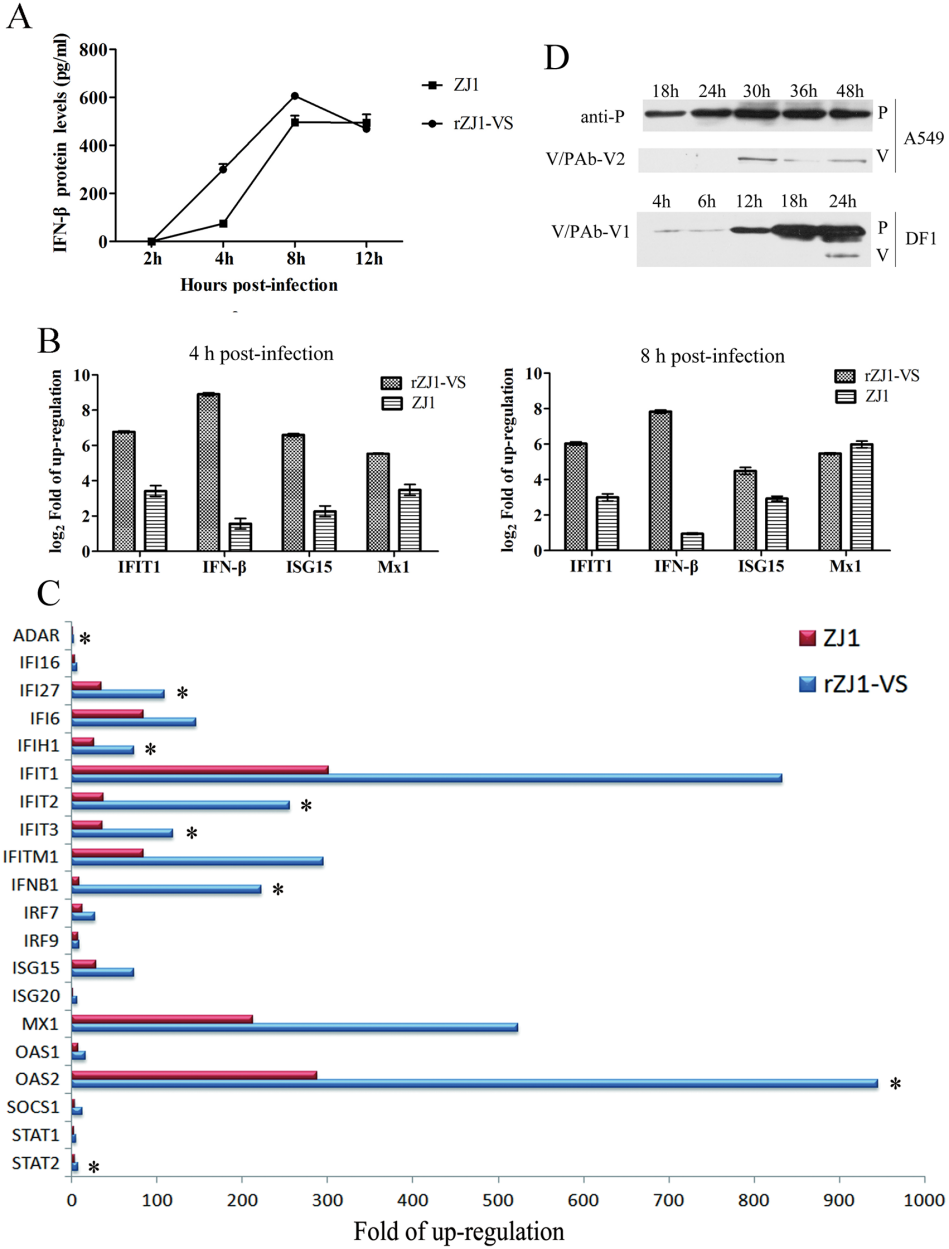
The expression levels of IFN-β and IFN-responsive genes in NDV-infected A549 and DF1 cells. (A) The IFN-β protein levels in A549 cells stimulated by NDV were assayed by ELISA so as to determine its effect to IFN signaling. (B) The IFN-responsive genes IFIT1, ISG15, Mx1, and IFN-β were further quantified by Real-time PCR in V-deficient rZJ1-VS and *wt* ZJ-1-infected A549 cells. (C) IFN-responsive genes in rZJ1-VS or ZJ1 infected DF1 cells. Gene expression was compared between DF1 cells infected with rZJ1-VS and *wt* virus ZJ1 at a MOI 3. *Gene expressions were enhanced significantly in rZJ1-VS-infected cells than those in rZJ1-infected cells (P < 0.01). (D) The V protein expression levels in NDV-infected A549 and DF1 cells. The P and V protein were detected in WB assay at different time points post infection with NDV strain ZJ1.

The tyrosine phosphorylation of STAT1 transcription factor by the *Janus* family of tyrosine kinase (JAK) enzymes plays central roles in mediating IFN-dependent biological responses, including induction of IFN-responsive genes [23]. Since NDV V protein mediates phospho-STAT1 degradation, the IFN-β protein levels stimulated by NDV were determined by ELISA so as to determine its effect to IFN signaling. First of all, the V-deficient rZJ1-VS induced much higher level of IFN-β protein than that induced by *wt* ZJ1 at the early period of infection (Fig 6A). Obviously, rZJ1-VS-infected cells have produced approximately 450 pg IFN-β into 1.5 mL media at 4 hpi, which was about 6 times higher than the level produced by *wt* ZJ-1 infected cells at the same time point. The IFN-responsive genes were further quantified and compared between V-deficient rZJ1-VS and *wt* ZJ-1-infected A549 cells. Both rZJ1-VS and ZJ1 induced the up-regulation of IFN downstream antiviral genes, including IFIT1, ISG15, Mx1, and IFN-β (Fig 6B), which was in correspondence with the IFN-β levels in the supernatant (Fig 6A). The deficiency of V protein in rZJ1-VS induced about 167.6, 10.6, 20.9 and 4.4 times higher levels of IFN-β, IFIT1, ISG15, and Mx1 in infected cells. To determine whether V-introduced degradation of phospho-STAT1 blocked IFN signaling in chicken-derived DF1 cells, we conducted PCR microarray experiments on IFN-responsive genes induced by NDV infection. Using RT²Profiler PCR Array Human Type I Interferon Response (Qiagen, PAHS-016Z), gene expression was compared between A549 cells infected with rZJ1-VS or the *wt* virus ZJ1 at a MOI of 3. Increased expression was shown for a number of IFN-stimulated genes such as IFNB1 (interferon, beta 1), IFIT1 (interferon-induced protein with tetratricopeptide repeats 1), OAS1 (2'-5'-oligoadenylate synthetase 1) and MX1 (myxovirus resistance 1, interferon-inducible protein p78), which are involved in cellular defense against viral infection (Fig 6C). Up-regulation is due to pattern recognition receptors (PRRs) recognizing viral components [44, 45].

IFN downstream antiviral genes were both induced by rZJ1-VS and ZJ1; however, the fold-change of those genes in rZJ1-VS-infected cells were significantly (P <0.01) higher than that in ZJ1-infected cells. ADAR, IFIH1, IFIT1-3, IFITM1, IRF7, IRF9, ISG15, ISG20, MX1 and OAS1 are downstream of IFN and have key roles in the antiviral response to RNA virus [46]. Microarray analysis showed that the levels of ADAR, IFIH1, IFIT2-3, Mx2 and OAS2 mRNA in both rZJ1-VS and ZJ1-infected cells increased by 5- to 30-fold at 6 hpi compared with mock-infected cells (Fig 6C). Compared with ZJ1-infected cells, rZJ1-VS-infected cells showed significantly elevated expression of 30 IFN-responsive genes by 2.5- to 1.5-fold (P < 0.05) at 6 hpi. This increase was seen in all tested genes downstream of IFN: ADAR, IFIH1, IFIT1-3, IFITM1, IRF7, IRF9, ISG15, ISG20, MX1 and OAS1. These results indicated that the absence of V CTD seriously impaired viral evasion of the IFN system.

Given that the V protein blocks IFN induction and apparently IFN signaling, and our observations showed that the *wt* virus induced high levels of ISGs in both A549 and DF1 cells, we further investigated the kinetic of the V protein expression in NDV infected cells. As shown in Fig 6D, the P protein was detected before 18 hpi, however, the V protein was under detectable level before 30 hpi in A549 cells and 24 hpi in DF1 cells, suggesting that the levels of expression of V protein in the infected cells is not sufficient to block IFN signaling at early times post infection and consequent induction of ISGs.

## Discussion

In the course of infection by a virus, the host establishes an antiviral state by activating IFN pathways and the viruses evolves mechanisms for IFN evasion [7, 8, 22, 47, 48]. STAT1, downstream of IFN, is considered a target for NDV to degrade, as observed in human 2fTGH cells [21]. However, we showed that total STAT1 expression was not degraded in NDV-infected Vero and A549 cells, which is similar to the previous report in DF-1 cells stably expressing NDV V protein [24]. In Vero cells, which are defective in IFN production [49], NDV infection did not lead to reduction of total STAT1.

Our results support that STAT1 expression was not degraded by NDV V protein in A549 and Vero cells. This result was further confirmed by transfection experiments. The amount of STAT1 was tested in cells expressing V proteins from LaSota (with a genomic RNA of 15,186 nt), ZJ1 (15,192 nt) and 9a5b (15,198 nt). When V protein from eukaryotic expression plasmids was over-expressed in cells, STAT1 protein was not degraded (Fig 1). Thus, our results showed co-existence of V proteins and STAT1 proteins in certain cells. This result suggested the mechanism is more complicated than V degradation of all STAT1 proteins. As reported in the researches on other paramyxoviruses, V proteins evade host cell IFN signal transduction not simply via degrading STATs [7]. The V protein of *Henipavirus*, namely, Nipah virus and Hendra virus, inhibits cellular responses to IFN through binding and cytoplasmic sequestration of both STAT1 and STAT2 [50, 51]. Measles Virus V Protein also does not degrade STATs but effectively prevents IFN-induced STAT1 and STAT2 nuclear import to inhibit IFN-induced transcriptional responses [52].

We further analyzed whether STAT1 phosphorylation patterns were altered in NDV-infected cells. An experimental model was first made to determine changes in phospho-STAT1. Phospho-STAT1 faded away in 2–4 h after stimulation, probably due to removal of cellular protein tyrosine phosphatase(s) [42, 43]. To exclude the interference of phosphatase(s) in the reduction of phosphor-STAT1, mock-infected and infected cells were stimulated with IFN-α before Western blots at different time points to ensure that STAT1 was phosphorylated. Treatment of IFN-α led to STAT1 phosphorylation in mock-infected cells. In contrast, accumulation of phospho-STAT1 was reduced by infection with NDV, which affected the level of total STAT1. Reduction of phospho-STAT1 was also observed in cells transfected with V-expressing plasmids compared to those in mock-infected cells after stimulation with IFN-α. These results showed that IFN-α was the prerequisite for V protein mediated STAT1 degradation, suggesting phospho-STAT1 rather than un-phosphorylated protein was the target for NDV V protein. It is well known that receptor-associated JAKs phosphorylate latent cytoplasmic STATs when cells are stimulated by IFN-I [22]. After phosphorylation, STAT1 and STAT2 form a heterotrimeric complex with an additional non-STAT protein, IRF-9 [22]. Phospho-STAT1 is assumed to be the target of NDV to degrade, since STAT1 was not degraded by NDV in normal cells or those cells stimulated with IFN-γ (Fig 2).

Since the reduction of phospho-STAT1 could be caused by degradation or dephosphorylation, we detected phospho-STAT1 in cells treated with an ubiquitin E1 inhibitor PYR-41 (Fig 3A and 3B)., It showed that phospho-STAT1 was not affected by NDV infection when an ubiquitin-related enzyme was inhibited, suggesting that reduction of phospho-STAT1 was caused by degradation by the ubiquitin-proteasome system. Further researches confirmed that V mediated STAT1 degradation was associated with its phosphorylation. Since latent cytoplasmic STAT1 proteins are phosphorylated on Tyr-701 by the JAK enzymes in response to stimulation by IFN-α, which is a critical step in IFN signaling [22, 23], the Tyr-701 in plasmid pFlag-STAT1 expressing a flag-tagged STAT1α protein was replaced by Phe to construct a dominant-negative STAT1α mutant [31]. After co-infection with NDV, the exogenous STAT1 expressed by plasmids were obviously degraded by NDV. In comparison, the expression level of dominant-negative STAT1 Y701 was not affected (Fig 3C), suggesting the phosphorylated STAT1 was the target for NDV. These results were consistent with the previous observations of SV5 and mumps virus, which target STAT1 for proteasome-mediated degradation [53–55].

Total STAT1 protein did not decrease as much as phospho-STAT1. This suggested that accumulation of STAT1 in NDV-infected cells was affected by two factors. One factor was that part of phospho-STAT1 was degraded by NDV as described above. Another factor was up-regulation of STAT1 mediated by host pattern recognition receptors (PRRs), especially mammalian retinoic acid-inducible gene I (RIG-I) [32, 47, 56–58]. Our results in RT²Profiler PCR Array confirmed that the STAT1 mRNA was significantly elevated after NDV infection (Fig 6C). This result indicated a balance between degradation and up-regulation of STAT1. In these cells expressing a large amount of STAT1 proteins, total STAT1 will not be obviously affected by phospho-STAT1 degradation; while in those cells expressing a small amount of STAT1 proteins, total STAT1 will be obviously affected. It is the reason why the level of total STAT1 differed in different cells infected with different NDV strains.

Our data clearly demonstrated that V protein was a major factor in degrading phospho-STAT1. In transfection experiments, plasmid-expressed V protein results in degradation of all phospho-STAT1 induced by IFN-α. To confirm this result, a V-deficient recombinant virus, rZJ1-VS, was recovered from a cDNA clone. To exclude the possibility that trace amounts of V protein were encoded via slipped-strand mispairing at late stages of infection, only results observed at early time points were analyzed and compared. Knocking-down V protein from NDV resulted in no degradation of phospho-STAT1 and seriously impaired the NDV evasion of the IFN system. The IFN-stimulated antiviral genes ADAR, IFIH1, IFIT1-3, IFITM1, IRF7, IRF9, ISG15, ISG20, MX1 and OAS1 in DF1 were expressed significantly higher than when induced by *wt* virus ZJ1. Similar results were observed in A549 cells. The V-deficient rZJ1-VS induced approximately 6 times higher levels of IFN-β than that produced by *wt* ZJ-1 infected cells at the same time point (Fig 6A). Correspondingly, rZJ1-VS, which expressed incomplete V protein, induced about 167.6, 10.6, 20.9 and 4.4 times higher levels of IFN-β, IFIT1, ISG15, and Mx1 in infected cells than that in wt ZJ1-infected A549 cells (Fig 6B). These results suggested that the CTD domain of V protein was an important component for NDV antagonization of IFN signaling.

In the real-time PCR assay and RT²Profiler PCR Array, *wt* ZJ1 induced high levels of ISGs at early period of infection in both A549 and DF1 cells, suggesting STAT1 signal transduction were not wholly inhibited. To ascertain the reason for this phenomenon, we detected the kinetics of the V protein expression in NDV-infected cells at different time points. It showed that the V protein was not detectable in DF1 and A549 cells before 30 hpi in A549 cells and 24 dpi in DF1 cells, however, the P protein was detected before 18 hpi (Fig 6D), suggesting that the levels V protein expression in the infected cells is not sufficient to block IFN signaling at early times post infection and consequent induction of ISGs.

Our results involving the virulence of the recombinant NDV lacking V protein indirectly supported this hypothesis. Retardation of rZJ1-VS in cells did not affect pathogenicity (Fig 4, Tables 3 and 4), suggesting the V protein is not essential for the propagation of VII genotype NDV even though it plays a role in IFN antagonism. This result was different from observations that the pathogenicity of V-deficient viruses is greatly weakened [15, 18, 19, 21]. However, all other V-deficient recombinant NDV strains belong to class I genotype II and were isolated in the mid-20th century. VII genotype stains, which are current prevalent in chicken and goose flocks [59], might have evolved additional anti-IFN strategies to facilitate propagation. In summary, we showed that in NDV-infected cells, phospho-STAT1 induced by IFN-α rather than unphosphorylated protein was targeted for degradation. We hypothesize that NDV antagonism of IFN signaling is both complex and economical. V protein, the major antagonist to IFN-I, is in the cytoplasm and degrades STAT1 as soon as STAT1 is phosphorylated and the CTD of V protein is critical for effective degradation of phospho-STAT1. The mechanisms described here for Newcastle disease added to knowledge about the detailed strategies that paramyxoviruses use to evade IFN.

## Supporting Information

S1 Fig The level of phosphorylated STAT1 after IFN-α-stimulation. A549 and Vero cells were incubated with 500 U/mL human IFN-α (Sigma) in 1 mL DMEM at 37°C for 15 min. The cells were then washed with PBS for three time, and cultured in DMEM containing 2% fetal bovine serum (FBS) at 37°C. The STAT1 and phospho-STAT1 were detected at 5 min, 15 min, 30 min and 1 h post infection. It was shown the phosphorylated STAT1 faded away in 1 h after stimulation, probably due to removal of cellular protein tyrosine phosphatase(s).
(TIF)
