# Supplementary figures and images for: Newcastle Disease Virus V Protein Targets Phosphorylated STAT1 to Block IFN-I Signaling
Source: PLoS One. 2016 Feb 9;11(2):e0148560. doi: 10.1371/journal.pone.0148560 (PMC4747598; doi:10.1371/journal.pone.0148560)

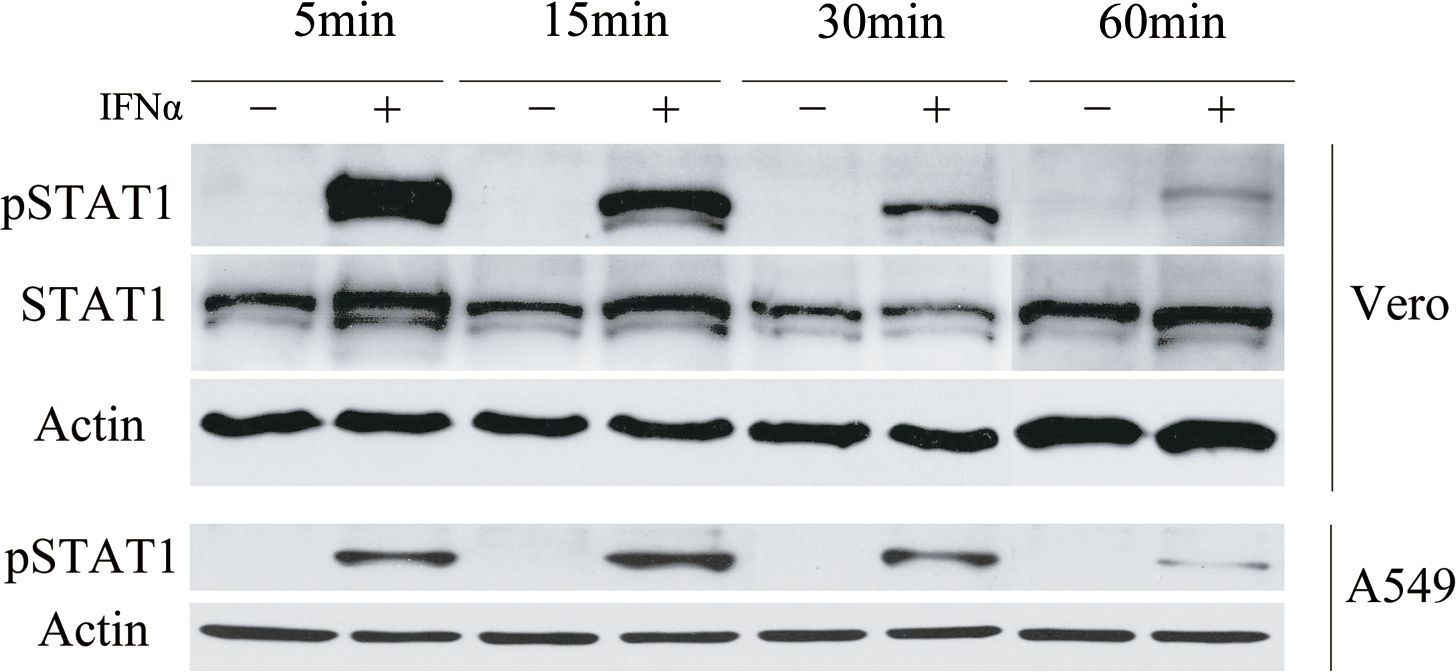

Supplement: S1 Fig — A549 and Vero cells were incubated with 500 U/mL human IFN-α (Sigma) in 1 mL DMEM at 37°C for 15 min. The cells were then washed with PBS for three time, and cultured in DMEM containing 2% fetal bovine serum (FBS) at 37°C. The STAT1 and phospho-STAT1 were detected at 5 min, 15 min, 30 min and 1 h post infection. It was shown the phosphorylated STAT1 faded away in 1 h after stimulation, probably due to removal of cellular protein tyrosine phosphatase(s). (TIF) [file pone.0148560.s001.tif]
